# Supplementary material for: The architecture and operating mechanism of a cnidarian stinging organelle
Source: Nat Commun. 2022 Jun 17;13:3494. doi: 10.1038/s41467-022-31090-0 (PMC9205923; doi:10.1038/s41467-022-31090-0)
Supplement: Supplementary file 1 — Supplementary Information [file 41467_2022_31090_MOESM1_ESM.pdf]

The architecture and operating mechanism of a cnidarian stinging organelle

Ahmet Karabulut, Melainia McClain, Boris Rubinstein, Keith Z. Sabin, Sean A. McKinney, Matthew C. Gibson

Supplementary Figures 1-7

Supplementary Note 1

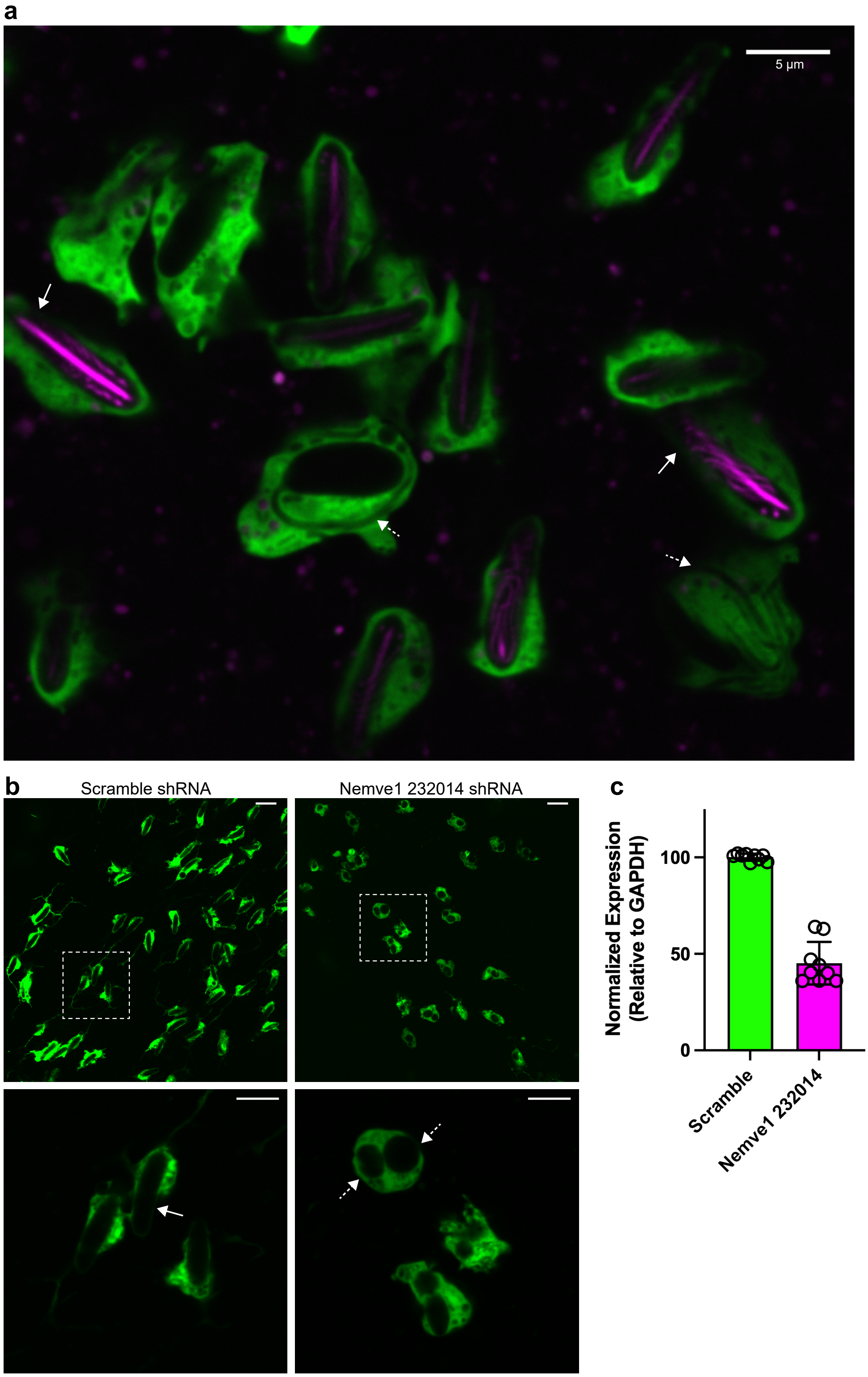

Supplementary Fig. 1 | TRITC incorporation into the nematocyst threads. a Nematocytes (green) of a transgenic *N. vectensis* primary polyp expressing EGFP under the control of the nematogalectin promoter. The EGFP expressing nematocytes with mature capsules containing TRITC labeled threads (arrows) and immature capsules with maturing threads (dashed arrows) were shown. (30 primary polyp body columns, 6 experiments). Scale bar 5 µm. **b** Effects of *Nemve1_232014* knockdown on nematocyte thread morphogenesis and capsule formation. Representative capsules (dashed boxes) of scramble shRNA control (*n*=0/14 primary polyps, arrow) and shRNA knockdown of *Nemve1_232014* showing abnormal capsules (*n*=17/17 primary polyps, dashed arrow). Scale bars 5 µm. **c** The transcript levels of *Nemve1_232014* after knockdown with scramble shRNA (*n*=9 technical replicates of three independent experiments; green) and shRNA targeting *Nemve1_232014* (*n*=9 technical replicates of three independent experiments; magenta). The plots show three independent knockdown experiments performed with ~200 polyps per sample, each sample was performed in triplicate, and measured once. Statistical significance was determined using a two-tailed, unpaired students t-test (*t*=14.72, CI [-62.82, -47.0], df=16 Cohen's *d*=6.9, p=1.0132E-10). Bar ± error bar = mean ± SD.

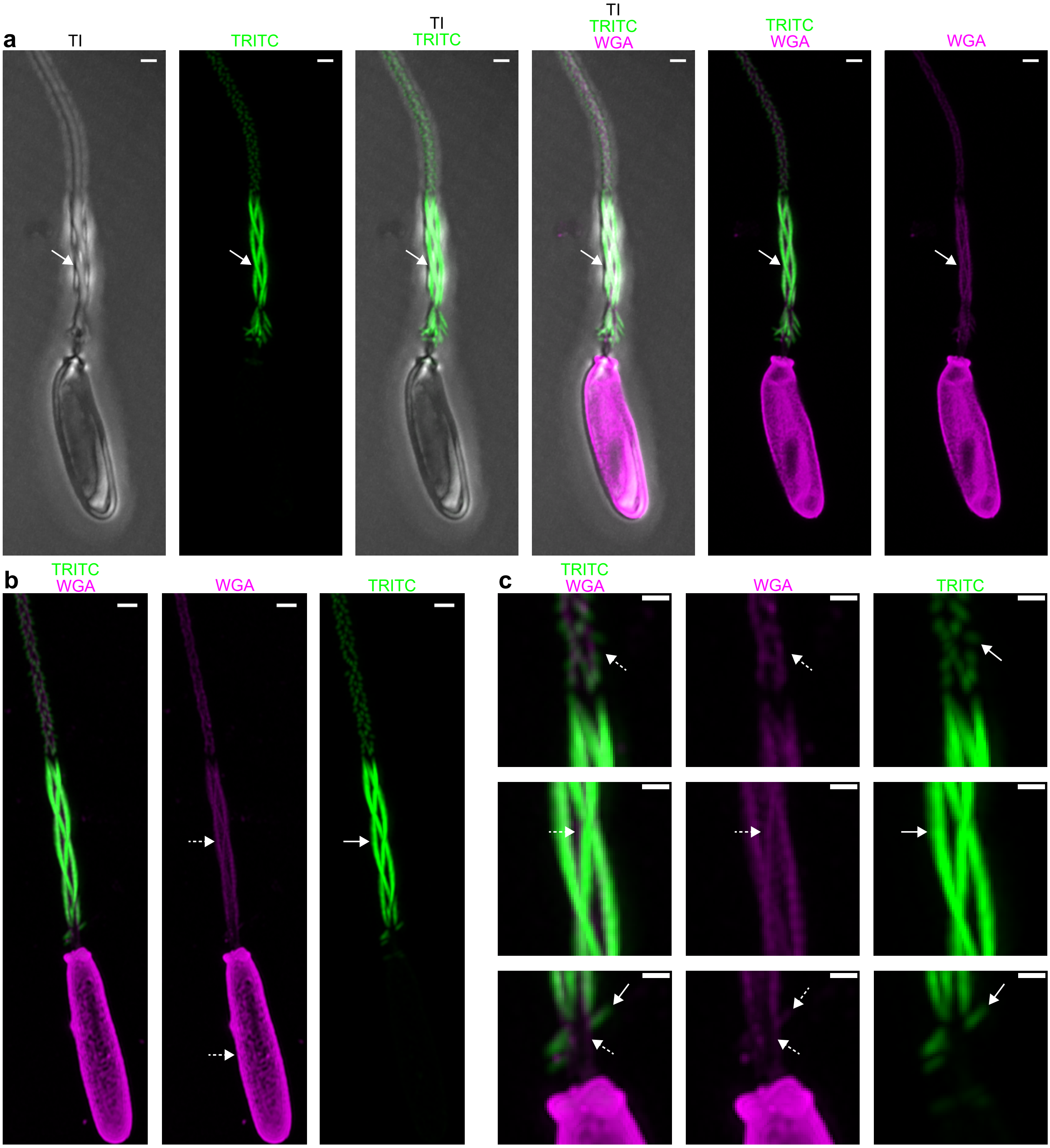

Supplementary Fig. 2 | TRITC and WGA co-staining of discharged capsules and threads. a Images of the capsule, shaft and a fraction of the tubule in Transillumination channel (TI), TRITC (green), WGA (magenta) and overlapping channels of a discharged thread. Arrows indicate overlapping of the shaft filament in each panel. Scale bar 0.5 µm. **b** Super resolution fluorescent image of a discharged thread showing TRITC labeled material (green, arrows) and WGA labeled material (magenta, dashed arrows in thread and capsules) of a discharged nematocyst. Scale bar 0.5 µm. **c** Magnified regions of panel b. The differential labeling of the thread by TRITC (green, arrows) and WGA (magenta, dashed arrows) in overlapping and individual channels in distinct sections of the thread. (Representative of purified and discharged threads from ~200 polyps). Scale bar 0.5 µm.

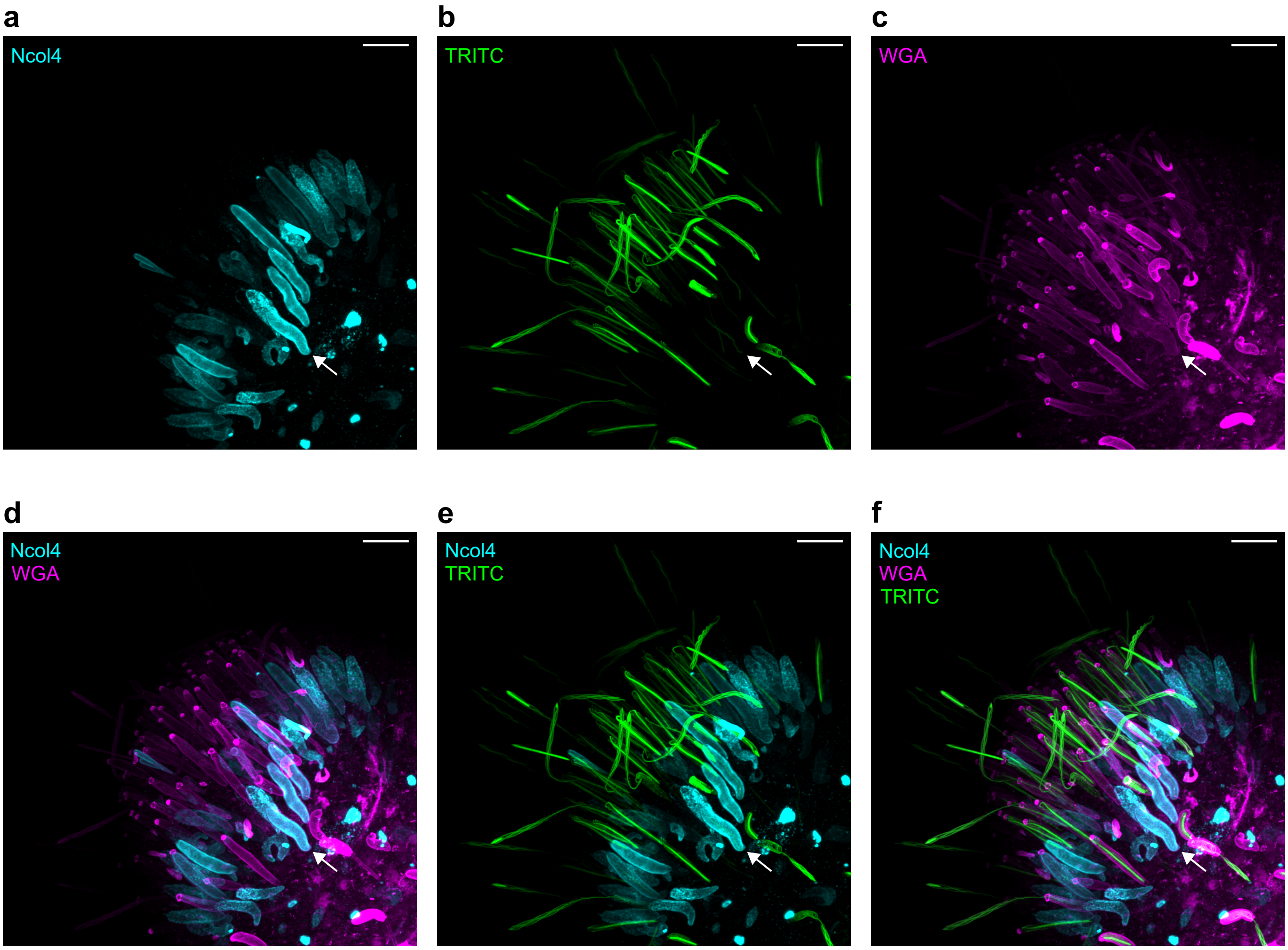

Supplementary Fig. 3 | Minicollagen Ncol4 antibody staining of discharged and undischarged capsules in a *Nematostella* tentacle. Images of the tentacle stained with Ncol4 antibody (cyan), TRITC (green) and WGA (magenta). Arrows indicate maturing nematocysts stained with Ncol4 antibody (Tentacle image represents n=3 immunostaining experiments). **a** Ncol4 (cyan) antibody staining. **b** TRITC (green). **c** WGA (magenta). **d** Combined channels of Ncol4 (cyan) and WGA (magenta). **e** Combined channels of Ncol4 (cyan) and TRITC (green). **f** Ncol4 (cyan), WGA (magenta) and TRITC (green). Scale bar 5 μm.

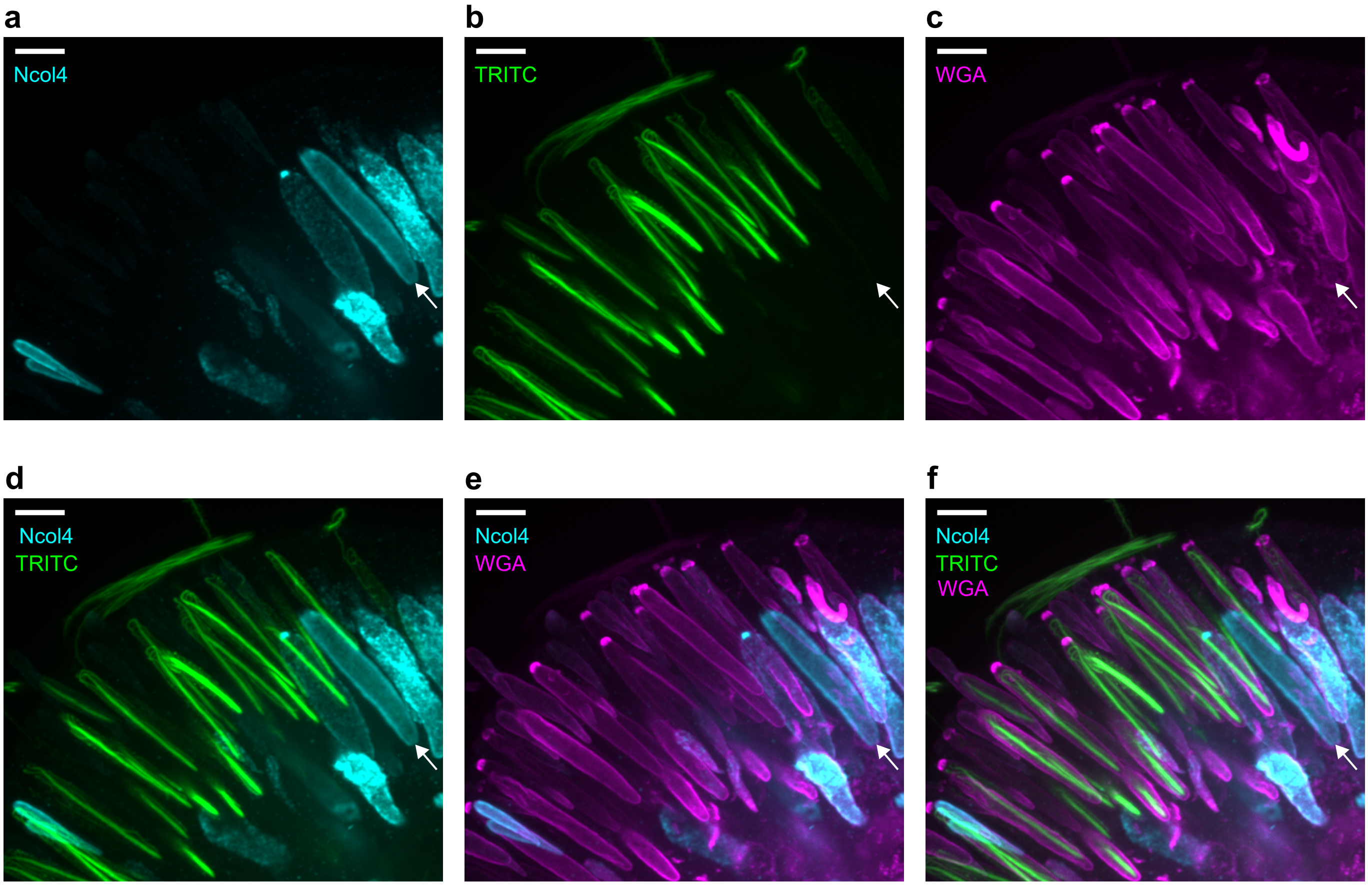

Supplementary Fig. 4 | A magnified region of the minicollagen Ncol4 antibody staining of the tentacle image shown in Supplementary Fig. 3. Images of the tentacle stained with Ncol4 antibody (cyan), TRITC (green) and WGA (magenta). Arrows indicate maturing nematocysts stained with Ncol4 antibody (Tentacle image represents *n*=3 immunostaining experiments). **a** Ncol4 (cyan) antibody staining. **b** TRITC (green). **c** WGA (magenta). **d** Combined channels of Ncol4 (cyan) and WGA (magenta). **e** Combined channels of Ncol4 (cyan) and TRITC (green). **f** Ncol4 (cyan), WGA (magenta) and TRITC (green). Scale bar 5 µm.

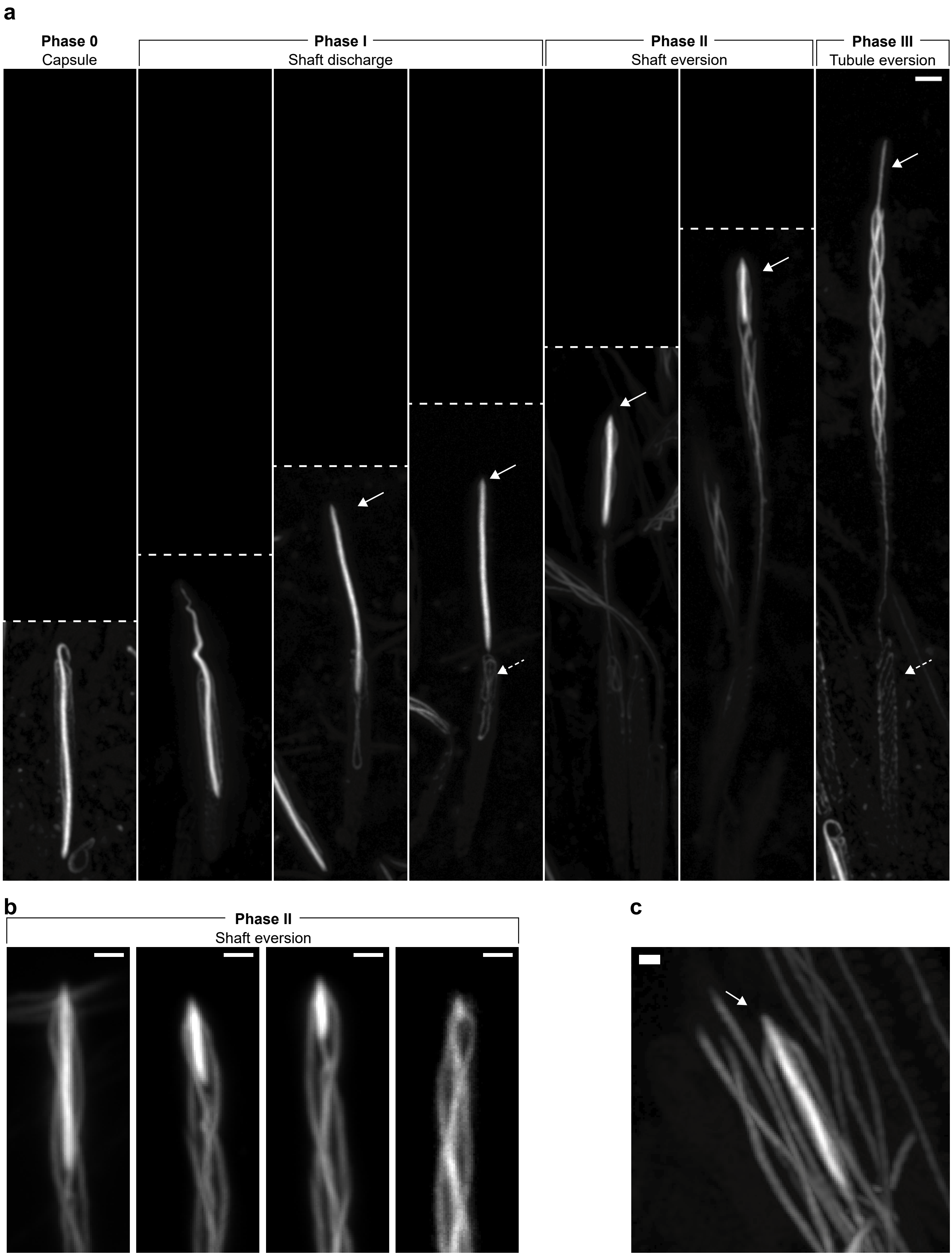

Supplementary Fig. 5 | The snapshots of threads captured during eversion. a The reconstruction of the sequence of events that occur during capsule discharge from still images (*n*=21 partially discharged threads from 4 primary polyp tentacles fixed with Lavdovski's reagent, 3 experiments). The snapshots of TRITC labeled everting thread shaft. The apical region of the shaft filament was seen as attached to the capsule. The tip of evaginating shaft (arrows) from the shaft's apex during the eversion process was shown. The tubule (dashed arrow) attachment to the basal end of the shaft filaments was shown while traversing through the everting shaft. The tubule lagging end was seen to remain inside the capsule (last panel, dashed arrow). Scale bar 1μm. **b** The snapshots of the tip of the evaginating TRITC labeled shaft filaments during the shaft eversion in Phase II. (*n*=20 partially discharged threads from 4 primary polyp tentacles) Scale bar 1μm. **c** Super-resolution image of the tip of the shaft filaments (arrow) during the shaft eversion in Phase II. Scale bar 1μm.

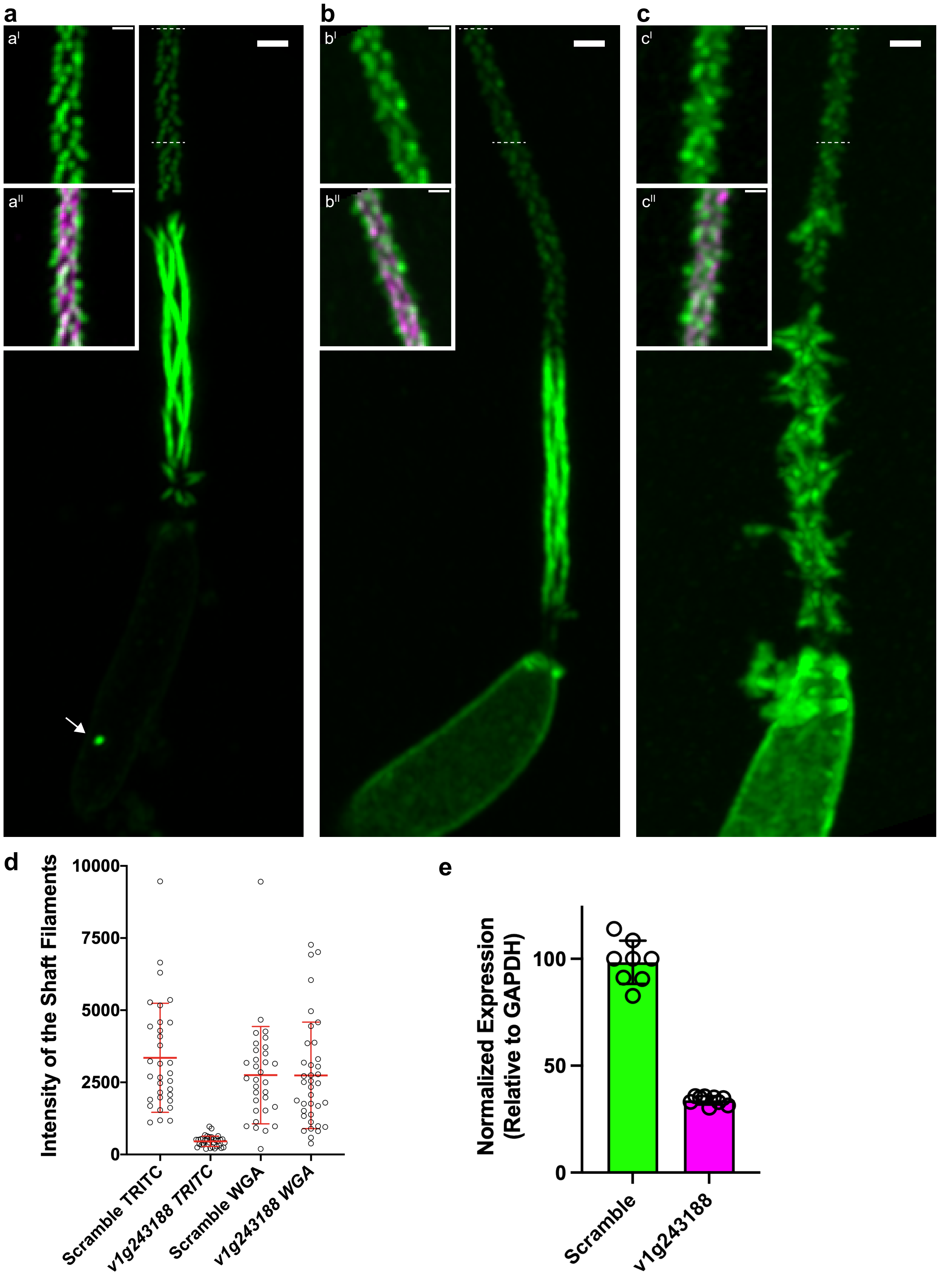

Supplementary Fig. 6 | Effects of *v1g243188* gene knockdown on the thread structures. a-c The structure of the shaft and the tubule after knockdown with shRNAs in TRITC labeled, discharged threads. (Representative images of purified and discharged threads of scramble and *v1g243188* knockdown samples, ~300 primary polyps per sample). Scale bars,1µm. Magnified regions of the tubule (dashed lines) were shown in insets, TRITC (green), WGA (magenta). Scale bars of insets, 500nm. **a** The structure of the shaft and the tubule after knockdown with scramble control shRNA. TRITC enriched bright dot inside the capsule was shown (arrow). **b** The structure of the shaft and the tubule after knockdown with *v1g243188* shRNA. **c** Strong phenotype showing delaminated shaft after knockdown with *v1g243188* shRNA. **d** Quantified TRITC and WGA-488 fluorescence signal intensity of the shaft filaments after knockdown with scrambled shRNA (n=32 individual threads imaged, purified and discharged from ~300 primary polyps electroporated with shRNA) and *v1g243188* shRNA (n=37 individual threads imaged, purified and discharged from ~300 primary polyps electroporated with shRNA). Images were taken at the same settings and background was subtracted. Statistical significance was determined using a two-tailed, unpaired students t-test. Values were in arbitrary units. TRITC (t=9.285, CI [-3515, -2271], df=67 Cohen's d=2.16, p=1.18E-13), WGA (t=0.01863, CI [-862.14, -846.4], df=67 Cohen's d=0.0045, p=0.98519). Line ± error bar = mean ± SD (red). **e** The relative transcript levels of *v1g243188* normalized to GAPDH after knockdown with scramble shRNA (n=8 technical replicates of three independent experiments; green) and shRNA targeting *v1g243188* (n=9 technical replicates of three independent experiments; magenta). Statistical significance was determined using a two-tailed, unpaired students t-test (t=18.83, CI [-71.86, -57.25], df=15 Cohen's d=8.9, p=7.54E-12). Bar ± error bar = mean ± SD.

a

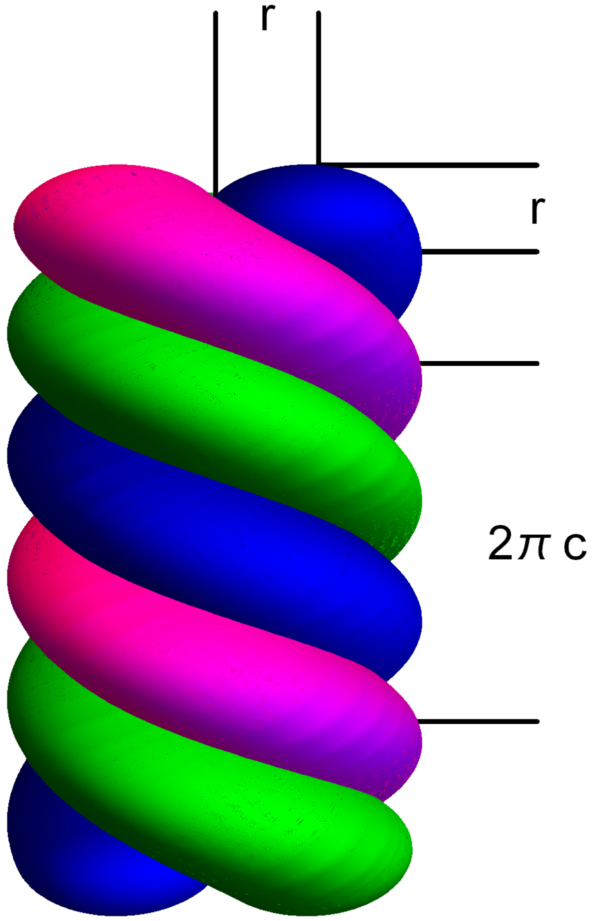

Supplementary Fig. 7 | A segment of tightly coiled shaft before eversion. The diameter of the shaft is equal to double diameter of the filament while the pitch *c* satisfies the condition $2\pi c = 6r$.

Supplementary Note 1

1 Helix elastic energy

Consider a filament of radius r bent into a helix (no twisting assumed to simplify calculations) with radius R and pitch c defined by the parametric equation

$$x = R\cos t, \quad y = R\sin t, \quad z = ct. \tag{1}$$

The curvature of such a helix is $\kappa = R/(R^2 + c^2)$. The bending energy e per unit length is given by [1]

$$e = \pi Y_0 \kappa^2 r^4/8, \tag{2}$$

where Y_0 stands for the filament Young modulus. For tightly coiled structure (its segment is shown in Supplementary Figure 7) we obtain that $2\pi c_c = 6r$ giving $c_c = 3r/\pi \approx r$. The radius of this coil $R_c = r$ and the curvature $\kappa_c = 1/(2r)$. Thus we find the bending energy E_c of the filament of the length L

$$E_c = \pi Y_0 L r^2/32 \approx Y_0 L r^2/10. \tag{3}$$

Consider an everted uncoiled helix with the increased radius $R_{uc} = kR_c = kr,\ k > 1$ and pitch $c_{uc} = mc_c = mr,\ m > 1$. Thus, the curvature of this helix reads $\kappa_{uc} = k/((k^2 + m^2)r) = 2k/(k^2 + m^2)\kappa_c$. This leads to the expression for the bending energy

$$E_{uc} = 4k^2 E_c/(k^2 + m^2)^2. \tag{4}$$

The measurements of the geometric parameters of the coiled and uncoiled helices produce the following values (in μm)

$$R_c = r = 0.12,\ R_{uc} = 0.6,\ c_c = r = 0.12,\ c_{uc} = 1.2,$$

leading to $k = 5$ and $m = 10$. Thus the energy stored in the uncoiled structure is less than 1% of that stored in the coiled structure. This estimate means that nearly all bending energy stored in the inverted structure $E_b = 3E_c$ is converted into kinetic energy $E_k = 3E_c$ that can be used for eversion of the shaft-tubule connector and part of the tubule.

2 Minicollagen fibers material parameters

The staining of the shaft filaments shows that they are made of Ncol1 and Ncol4 in approximately equal proportions. The structural analysis of these proteins exhibits CRD regions, polypropiline segment(s) and more flexible collagen motif. Similar structure is also observed in the CPP-1 molecule found in *Hydra* nematocyst that has Young modulus of $Y = 7.8 \pm 8.0$ MPa in the bulk [2]. Collagen fibers demonstrate Young modulus values order of few hundreds MPa found using different methods [3, 4]. It is convenient to use Y as an estimate for Y_0. This gives us an estimate for E_k for the filaments of the length $L = 20\ \mu$m

$$E_k = 3E_c = 0.3YLr^2 = 1\ pJ.$$

3 Energy cost of eversion

To estimate the energy cost of this process one has to consider the connector as an elastic cylinder of radius r and length $\mathcal{L}$ everting inside out. With some assumptions about the tubule elastic properties such a problem has an exact solution for the eversion work A_e that reads

$$A_e = \pi\mu\mathcal{L}r^2(1-\delta^2),$$

where μ is the is the shear modulus of the material and δ is the ratio of the inner cylinder radius to the outer one. Taking $\delta = 0.8$ and using shear modulus of the collagen $\mu = 35\ MPa$ we find

$$A_e = 50\mathcal{L}r^2 \cdot 10^{-12}\ J,$$

where the cylinder length $\mathcal{L}$ and radius r are measured in microns. Using here $r = 0.1$ we find $A_e = 0.5\mathcal{L} \cdot 10^{-12}\ J$. The length of the connector is $\mathcal{L} = 5\ \mu m$ and we finally arrive at $A_e = 2.5\ pJ$.

We observe that this energy is comparable to that of provided by the elastic energy of the shaft so that partial eversion of the connector is possible.

Supplementary References

[1] Landau LD, Lifshitz EM, *Theory of Elasticity*, Pergamon Press, 1970.

[2] Bentele T, Amadei F *et al*, New Class of Crosslinker-Free Nanofiber Biomaterials from Hydra Nematocyst Proteins, Sci.Reports, 2019, 9:19116.

[3] Dutov P, Antipova O *et al*, Measurement of Elastic Modulus of Collagen Type I Single Fiber, PLoS ONE, 2016, 11(1): e0145711.

[4] Van der Rijt JAJ, van der Werf KO *et al*, Micromechanical Testing of Individual Collagen Fibrils, Macromol. Biosci., 2006; 6: 697702.
