# Supplementary material for: The architecture and operating mechanism of a cnidarian stinging organelle
Source: Nat Commun. 2022 Jun 17;13:3494. doi: 10.1038/s41467-022-31090-0 (PMC9205923; doi:10.1038/s41467-022-31090-0)
Supplement: Supplementary file 3 — Description of Additional Supplementary Files [file 41467_2022_31090_MOESM3_ESM.pdf]

File name: Supplementary Movie 1

Description: Time lapse of TRITC dye (Magenta) incorporation inside the capsule of a nematocyte expressing EGFP (Green).

File name: Supplementary Movie 2

Description: Serial SEM of the longitudinal cross section of a basitrichous isorhiza.

File name: Supplementary Movie 3

Description: 3D reconstruction of the longitudinal cross section of a basitrichous isorhiza showing the connector regions (yellow), the shaft (blue) and a segment of the tubule (magenta) inside the capsule.

File name: Supplementary Movie 4

Description: Serial SEM of the traverse cross section of a basitrichous isorhiza.

File name: Supplementary Movie 5

Description: Time lapse of the discharge and eversion of a long basitrichous isorhiza in TRITC labeled polyps. Arrow indicate the capsule embedded inside the tissue.

File name: Supplementary Movie 6

Description: Discharge events recorded in TRITC labeled polyps in 5msec intervals.

File name: Supplementary Movie 7

Description: Time lapse of the discharge and partial eversion of a short basitrichous isorhiza in TRITC labeled polyp.

File name: Supplementary Movie 8

Description: Serial SEM of an everted shaft and the traversing uneverted tubule.

File name: Supplementary Movie 9

Description: Serial SEM of the longitudinal cross section of an everting thread showing uneverted tubule traversing inside the everted segments.

File name: Supplementary Movie 10

Description: Time lapse of the movement of a thread possibly blocked by an obstacle showing the bending of the tubule in 180 degree turns.

File name: Supplementary Movie 11

Description: Time lapse of the discharge and eversion of a short basitrichous isorhiza showing arrested tubule eversion and the forward movement of the uneverted tubule inside the everted fraction.
