# Supplementary material for: The architecture and operating mechanism of a cnidarian stinging organelle
Source: Nat Commun. 2022 Jun 17;13:3494. doi: 10.1038/s41467-022-31090-0 (PMC9205923; doi:10.1038/s41467-022-31090-0)
Supplement: Supplementary file 15 — Reporting Summary [file 41467_2022_31090_MOESM15_ESM.pdf]

nature portfolio

Corresponding author(s): Matthew C. Gibson

Last updated by author(s): Apr 25, 2022

Reporting Summary

Nature Portfolio wishes to improve the reproducibility of the work that we publish. This form provides structure for consistency and transparency in reporting. For further information on Nature Portfolio policies, see our Editorial Policies and the Editorial Policy Checklist.

Statistics

For all statistical analyses, confirm that the following items are present in the figure legend, table legend, main text, or Methods section.

n/a	Confirmed	
☐	☒	The exact sample size (n) for each experimental group/condition, given as a discrete number and unit of measurement
☐	☒	A statement on whether measurements were taken from distinct samples or whether the same sample was measured repeatedly
☐	☒	The statistical test(s) used AND whether they are one- or two-sided<br>*Only common tests should be described solely by name; describe more complex techniques in the Methods section.*
☒	☐	A description of all covariates tested
☒	☐	A description of any assumptions or corrections, such as tests of normality and adjustment for multiple comparisons
☐	☒	A full description of the statistical parameters including central tendency (e.g. means) or other basic estimates (e.g. regression coefficient) AND variation (e.g. standard deviation) or associated estimates of uncertainty (e.g. confidence intervals)
☐	☒	For null hypothesis testing, the test statistic (e.g. F, t, r) with confidence intervals, effect sizes, degrees of freedom and P value noted<br>*Give P values as exact values whenever suitable.*
☒	☐	For Bayesian analysis, information on the choice of priors and Markov chain Monte Carlo settings
☒	☐	For hierarchical and complex designs, identification of the appropriate level for tests and full reporting of outcomes
☐	☒	Estimates of effect sizes (e.g. Cohen's d, Pearson's r), indicating how they were calculated

Our web collection on statistics for biologists contains articles on many of the points above.

Software and code

Policy information about availability of computer code

Data collection	SmartSEM 6.00, Atlas 5.2.2.15
Data analysis	Fiji (ImageJ) (version 2.1.0/1.53c) was used for image processing. IMOD 4.9.10 was used for the analysis of EM samples. Graphpad Prism (9.3.1) was used for statistical analysis, Wolfram Mathematica (12.0) was used for energy estimates and modeling, Blender (2.93) and Adobe Photoshop 2021 was used for modeling , Adobe Adobe Illustrator 2021 was used for the preparation of the figures of this article.

For manuscripts utilizing custom algorithms or software that are central to the research but not yet described in published literature, software must be made available to editors and reviewers. We strongly encourage code deposition in a community repository (e.g. GitHub). See the Nature Portfolio guidelines for submitting code & software for further information.

Data

Policy information about availability of data

All manuscripts must include a data availability statement. This statement should provide the following information, where applicable:

- Accession codes, unique identifiers, or web links for publicly available datasets
- A description of any restrictions on data availability
- For clinical datasets or third party data, please ensure that the statement adheres to our policy

The main data supporting the results of this study are available within the article and the supplementary files of this article. Source data available for Supplementary Figures 1d. 6c and d. Original data underlying this manuscript can be accessed from the Stowers Original Data Repository at http://www.stowers.org/research/publications/libpb-1684

Field-specific reporting

Please select the one below that is the best fit for your research. If you are not sure, read the appropriate sections before making your selection.

☒ Life sciences ☐ Behavioural & social sciences ☐ Ecological, evolutionary & environmental sciences

For a reference copy of the document with all sections, see nature.com/documents/nr-reporting-summary-flat.pdf

Life sciences study design

All studies must disclose on these points even when the disclosure is negative.

Sample size	No sample size calculation was performed. For descriptive analysis, the images were acquired from minimum of ten independent spawning events each containing more than 300 animals from which tentacles and body columns were imaged to visualize nematocytes and TRITC labeled or unlabeled, discharged and undischarged nematocysts. The nematocysts capsules and threads were imaged in situ or after purification from independent spawns with identical results. Knockdowns were performed in three independent experiments each containing approximately 300 animals for qPCR.
Data exclusions	No data were excluded.
Replication	All attempts at replication were successful. All experiments were repeated with at least two or three independent trials. The number of replications is given in the figure legends and methods section.
Randomization	Embryos for shRNA injection were allocated at random between experimental and control groups. Animals used for descriptive analysis of cnidocyte architecture were selected at random.
Blinding	Blinding was not necessary for the primarily descriptive aspects of this work, and was not used to analyze shRNA knockdown samples in this study.

Reporting for specific materials, systems and methods

We require information from authors about some types of materials, experimental systems and methods used in many studies. Here, indicate whether each material, system or method listed is relevant to your study. If you are not sure if a list item applies to your research, read the appropriate section before selecting a response.

Materials & experimental systems

n/a	Involved in the study	
☐	☒	Antibodies
☒	☐	Eukaryotic cell lines
☒	☐	Palaeontology and archaeology
☐	☒	Animals and other organisms
☒	☐	Human research participants
☒	☐	Clinical data
☒	☐	Dual use research of concern

Methods

n/a	Involved in the study	
☒	☐	ChIP-seq
☒	☐	Flow cytometry
☒	☐	MRI-based neuroimaging

Antibodies

Antibodies used	Alexa Fluor 647 antibody (Goat anti-rabbit IgG (H+L), dilution 1:500, Thermo Fisher Cat# A21245, Lot 1981173);<br><br>Anti-NvNcol-4 antibody raised against Nematostella Vectensis minicollagen NvNcol-4 protein (Rabbit, dilution 1:500, kind gift from Suat Ozbek, Heidelberg University);<br><br>WGA-Oregon Green-488 conjugate (Dilution 1:500, Invitrogen Cat# W7024B Lot 2298084)<br><br>Rabbit anti-Ncol4 antibody was a kind gift from Dr. Suat Ozbek, Heidelberg University. Zenkert, C., Takahashi, T., Diesner, M. O. & Ozbek, S. Morphological and molecular analysis of the Nematostella vectensis cnidom. PLoS One 6, e22725 (2011).
Validation	The Rabbit-anti-NvNcol4 antibody was validated in Figure 5 I-L and 6 of the article from Zenkert, C., Takahashi, T., Diesner, M. O. & Ozbek, S. Morphological and molecular analysis of the Nematostella vectensis cnidom. PLoS One 6, e22725 (2011). We also validated this antibody by immunostaining and imaging of tentacles from independent staining experiments.<br><br>Goat anti-Rabbit IgG(H+L) Alexa Fluor 647 conjugated secondary antibody was validated for immunostaining by the vendor using HeLa cells stained with Rabbit anti- alpha Tubulin polyclonal antibody. We also validated this antibody in independent immunostaining experiments in vivo.

Animals and other organisms

Policy information about studies involving animals; ARRIVE guidelines recommended for reporting animal research

Laboratory animals	Laboratory strain of male and female Nematostella vectensis and the transgenic reporter lines generated from this laboratory strain were used in this study.
Wild animals	The study did not involve wild animals.
Field-collected samples	The study did not involve samples collected from the field.
Ethics oversight	No ethical approval, ethics oversight or guidance are required for the experiments involving cnidarians including Nematostella vectensis.

Note that full information on the approval of the study protocol must also be provided in the manuscript.
